# The assessment of bone health in children with juvenile idiopathic arthritis; comparison of different imaging-based methods

**DOI:** 10.1186/s12969-024-01018-7

**Published:** 2024-08-29

**Authors:** Thomas Augdal, Oskar Angenete, Pia Zadig, Anette Lundestad, Ellen Nordal, Xieqi Shi, Karen Rosendahl

**Affiliations:** 1 Section of Paediatric Radiology, University Hospital of North Norway, Tromsø, Norway; 2 Department of Clinical Medicine, Faculty of Health Sciences, UiT The Arctic University of Norway, Tromsø, Norway; 3 Department of Radiology and Nuclear Medicine, St Olavs Hospital, Trondheim, Norway; 4 Department of Circulation and Medical Imaging, Norwegian University of Science and Technology, Trondheim, Norway; 5 Department of Paediatrics, St Olavs Hospital, Trondheim, Norway; 6 Department of Paediatrics, and Research Group for Child and Adolescent Health, Department of Clinical Medicine, University Hospital of North Norway, UiT The Arctic University of Norway, Tromsø, Norway; 7 Department of Clinical Dentistry, The Faculty of Medicine, University of Bergen, Bergen, Norway; 8 Department of Oral and Maxillofacial Radiology, Faculty of Odontology, University of Malmö, Malmö, Sweden

**Keywords:** Osteoporosis, Child, Radiography, Cone-beam computed tomography, Dual-energy X-ray absorptiometry

## Abstract

**Background:**

Osteoporosis is increasingly being recognized in children, mostly secondary to systemic underlying conditions or medication. However, no imaging modality currently provides a full evaluation of bone health in children. We compared DXA, a radiographic bone health index (BHI (BoneXpert) and cone-beam CT for the assessment of low bone mass in children with juvenile idiopathic arthritis (JIA).

**Methods:**

Data used in the present study was drawn from a large multicentre study including 228 children aged 4–16 years, examined between 2015 and 2020. All had a radiograph of the left hand, a DXA scan and a cone-beam CT of the temporomandibular joints within four weeks of each other. For the present study, we included 120 subjects, selected based on DXA BMD and BoneXpert BHI to secure values across the whole range to be tested.

**Results:**

One hundred and twenty children (60.0% females) were included, mean age 11.6 years (SD 3.1 years). There was a strong correlation between the absolute values of BHI and BMD for both total body less head (TBLH) (*r* = 0.75, *p* < 0.001) and lumbar spine (L1-L4) (*r* = 0.77, *p* < 0.001). The correlation between BHI standard deviation score (SDS) and BMD TBLH Z-scores was weak (*r* = 0.34) but significant (0 = 0.001), varying from weak (*r* = 0.31) to moderate (*r* = 0.42) between the three study sites.

Categorizing BHI SDS and DXA BMD Z-scores on a 0–5 scale yielded a weak agreement between the two for both TBLH and LS, with w-kappa of 0.2, increasing to 0.3 when using quadratic weights. The agreement was notably higher for one of the three study sites as compared to the two others, particularly for spine assessment, yielding a moderate kappa value of 0.4 – 0.5.

For cone-beam CT, based on a 1–3 scale, 59 out of 94 left TMJ’s were scored as 1 and 31 as score 2 by the first observer vs. 87 and 7 by the second observer yielding a poor agreement (kappa 0.1).

**Conclusions:**

Categorizing DXA LS and automated radiographic Z-scores on a 0–5 scale gave a weak to moderate agreement between the two methods, indicating that a hand radiograph might provide an adjuvant tool to DXA when assessing bone health children with JIA, given thorough calibration is performed.

## Background

Osteoporosis is increasingly being recognized in children, mostly secondary to systemic underlying conditions or medications, such as inflammatory, hematological and oncological disorders, as well as malnutrition and immobilization [1, 2]. The diagnosis is based on a significant fracture history, and a dual-energy X-ray absorptiometry (DXA) Z-score < -2 standard deviations (SD), except for those presenting with a low-energy vertebral fracture in whom the fracture suffices [3]. Despite significant shortcomings, DXA is the most commonly used method for assessing bone mass in children, preferring the total body less head (TBLH) and lumbar spine (LS, 1-4) locations although alternative skeletal sites can be used when these are not feasible [2–5]. DXA-derived values for children are expressed as age-specific and sex-specific Z-scores. Normative paediatric data must be used for Z-score calculation, which are available for children older than 3 years for the TBLH, while LS measurements are available also for children aged <3 years [6–9].

DXA in children, with the assessment of areal bone mineral density (aBMD) is, however, flawed with biases. Firstly, DXA measurements underestimate BMD (in g/cm2) in children with short stature or growth /pubertal delay, thus, adjustment for bone age, or for skeletal size is mandatory. To adjust for skeletal size, volumetric BMD (vBMD or bone mineral apparent density [BMAD], in g/cm3) is calculated or BMD Z-scores are adjusted for height [10, 11] or for bone age [12] . Next, there is significant variation between DXA machines, more so for the LS and TBLH-locations than for hips [4]. And thirdly, the age- and sex-specific Z-score reference values available differ between different reference databases. Findings from a recent study showed that spine BMD Z-scores can vary by as much as 2 standard deviations (SD) depending on the normative database that is used to generate the Z-scores [13]. Moreover, DXA cannot differentiate between cortical and trabecular bone, hampering comparison to alternative methods.

Another imaging based technique, peripheral QCT (pQCT), assess cortical and trabecular bone separately, providing information on bone geometry not assessible with DXA [14]. Its main limitations are related to the need of proper positioning of the patient to achieve reproducibility, and to movements during the scan resulting in artefacts. Whether or not pQCT measurements adequately refect the whole skeleton, including the spine, is still under debate. Again, reference data are available, but have their limitations [15].

Except for a radiograph of the lateral spine to identify silent vertebral fractures, the role of radiography in the diagnosis of osteoporosis has been questioned, in part due to suboptimal image quality following the introduction of digital imaging. However, recent advances in detector technology has improved image quality significantly. Moreover, evaluation of bone structure has been, and still is an important part of the radiological assessment in a variety of diseases, such as skeletal dysplasias, metabolic and inflammatory disease, amongst others [16]. In 2009, an automated, digitized radiography (DXR)-based method for assessing peripheral bone geometry and density was introduced, measuring a cortical index (Bone Health Index, BHI) by hand radiographs in children over the age of 2-3 years [17, 18]. The method was recently extended down to newborns, based on data from 410 healthy children born in Paris in 1955 [19]. Although BHI assesses metacarpal cortex alone, while DXA assesses cortex and trabecular bone, several studies have demonstrated an association between the BHI and BMD as measured by DXA [20–26]. In a recent systematic review, seven papers compared digital X-ray radiogrammetry (DXR) with DXA, of which four found a moderate to strong degree of correlation [27]. However, two studies found a poor correlation, underscoring the need for further research.

In adults, panoramic radiography and CBCT have been used for the assessment of bone health. The mandibular cortical index (MCI), developed for panoramic radiography in 1994 by Klemetti et al [28], is a qualitative index assessing the appearances of the mandibular endostium [29]. Two recent systematic reviews, one based on panoramic radiography [29] and one on CBCT [30], concluded that both methods might represent auxillary tools for identifying postmenopausal females at risk of low bone mineral density. In children no similar studies are published.

Irrespective of the underlying cause, the radiographic appearances of osteoporosis are those of increased radiolucency and cortical thinning [1, 31, 32]. In young children the appearances of the zone of provisional calcification (ZPC) abutting the metaphysis, may provide additional information [33, 34].

The effective radiation dose for each of the imaging based methods discussed in this paper is low as compared to the annual background radiation dose of 3-5 milliSievert (mSv), ranging from 0.001-0.003 mSv for DXA (https://hps.org/publicinformation/ate/q13693.html), 0.06mSv for a hand radiograph (https://hps.org/physicians/documents/Doses_from_Medical_X-Ray_Procedures.pdf) to 0.02-0.5 mSv for a CBCT [35].

Routine follow-up of children with JIA often includes assessment of bone health [36]. We aimed to compare different techniques for the assessment of osteoporosis/low bone mass, namely DXA (WBLH, LS), bone health index (BHI) as assessed by the program BoneXpert, subjective assessment of a left hand radiograph (cortical thickness 2-4^th^ metacarpals, conspicuity of the zone of provitional alcification (ZPC) on a 0-2 scale, and cone-beam computed tomography (CBCT) of the mandible.

## Methods

Data used in the present study was drawn from a large multicentre study, the NorJIA study (www.norjia.com), including 228 children and adolescents with juvenile idiopathic arthritis (JIA) aged 4-16 years, examined between March 2015 and November 2020. As per protocol, all had a radiograph of the left hand and wrist, a DXA scan and a CBCT of the TMJs within 4 weeks of each other. For the present study, we included 120 subjects (>99% Caucasian) selected based on DXA BMD or BHI values, securing values across the whole range to be tested (balanced dataset).

The DXA scans were performed on a Lunar iDXA (Bergen, Trondheim) or Lunar Progidy (Tromsø) (both GE Lunar Corporation, Madison, WI). Age- and sex specific BMD Z-scores for posterior-anterior LS (L1-L4) and total body less head (TBLH) were calculated using Lunar iDXA enCore versjon 17 software and normative reference databases in accordance with the International Society for Clinical Densitometry (ISCD) pediatric official position [11]. Analyses were performed using bone-age adjusted BMD-values for children with a growth delay, defined as skeletal age < -2SD for age/gender, as assessed by the program BoneXpert [11, 37].

The radiographs of the left hand were taken with either a Acroma Triathlon T3 Precision system and a CXDI-810CW Canon detector with a pixel size of 125 µm at UNN; Carestream DRX evolution+ (USA) or Philips Digital Diagnost with a Canon detector in Bergen and Philips Digital Diagnost C 90 system with a SkyPlate detector, pixel size 148 µm at St Olavs. The focus-detector-distance was 115 cm and the focal spot size was 0.6mm at all three sites. All radiographs were exported to the program BoneXpert (Standalone version 3.2.1.12) Visiana, Holte, Denmark) and bone health index (BHI) and bone age (adjusted for sex and ethnicity) were registered together with demographic data. The formula used is BHI = π × (1 − T/W)/(LW)^0.33^. T is defined as the cortical thickness of the three middle metacarpals, W is the metacarpal width, and L is the bone length. The BoneXpert automatically compares the BHI to a Caucasian reference population with the same sex and converts it to a Z-score adjusted for bone age, the BHI SDS [38].

**Fig. 1:**
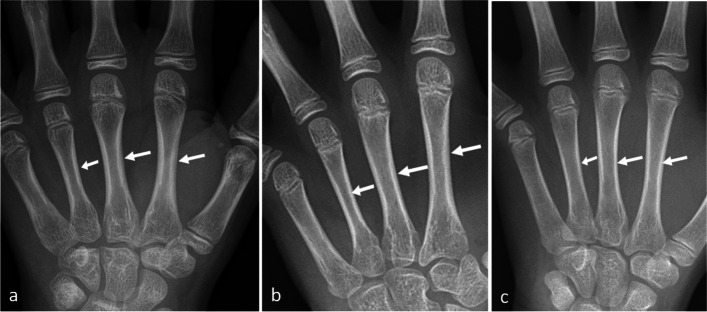
**a**-**c** Radiographs of the wrist in 3 females aged 10-11 years. Cortical thickness based on metacarpals 2-4 (arrows) was assessed using BoneXpert BHI Standard deviation score (SDS), classified on a 0-5 scale, exemplified by (**a**) SDS of +1.8, (**b**) SDS of 0 and (**c**) SDS of -2.4

**Fig. 2:**
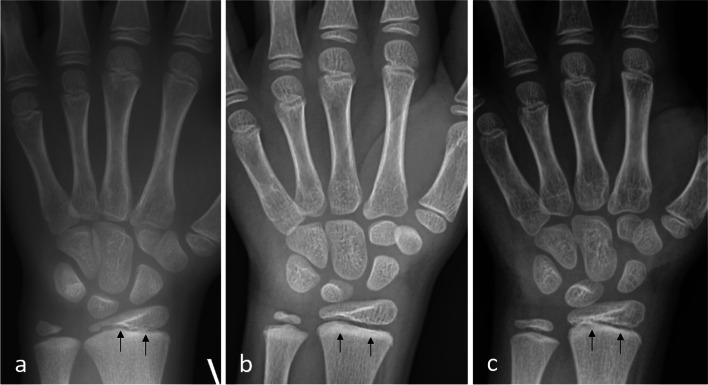
**a**-**c** Radiographs of the wrist in 3 females aged 6-7 years. Conspicuity/visibility of the zone of provisional calcification in radius only (ZPC) was scored as (**a**) not visible, (**b**) moderately  visible or (**c**) mildly  visible

In addition, cortical thickness, based on metacarpals 2-4, was scored subjectively on a continuous -4 to +4 scale (with one decimal; representing a “BHI-SDS equivalent”), by two experienced paediatric radiologists in consensus (>30 and 13 years of experience in pediatric radiology, respectively), knowing sex and bone age (Fig. 1a-c). We also scored subjective impression of bone structure (normal bone mass, suspected low bone mass or low bone mass), irregularity of the cortex (yes/no) and conspicuity/visibility of the zone of provisional calcification, radius only (ZPC) on a 0-2 scale (Fig. 2a-c).

Before scoring was performed, an atlas of left-hand radiographs with BHI varying from -4 to +4, and for males and females separately, was established. High resolution PACS screens were used for the assessment of radiographs.

CBCT volumes of the 120 subjects were selected and randomly coded. Image acquisition were performed employing either of three CBCT scanners with machine settings kVp / mAs / field of view (mm) / voxel dimension (isotropic, mm): 3D Accuitomo 170 (Morita Mfg Corp, Kyoto, Japan) 85 / 175 / 40*40*40 / 0.08; Promax 3D (Planmeca Oy, Helsinki, Finland) 90 / 13.6 / 200*200*60 / 0.40; or Scanora 3D (Soredex, Tuusula, Finland) 90 / 45 / 60*60*60 / 0.13, with the participants positioned in the Frankfort plane horizontal with their teeth in maximal intercuspal position.

Each CBCT data set was assessed independently by two specialists with more than 10 years of experience in interpreting TM-joints; one in medical radiology (OA) and the other in dento-maxillofacial radiology (Xie). Viewing took place under standardised conditions with dimmed light. The screen display was selected to one of the monitors default settings, DICOM mode. The observers were allowed to adjust the grey level, and contrast according to their individual preferences during viewing.

**Fig. 3:**
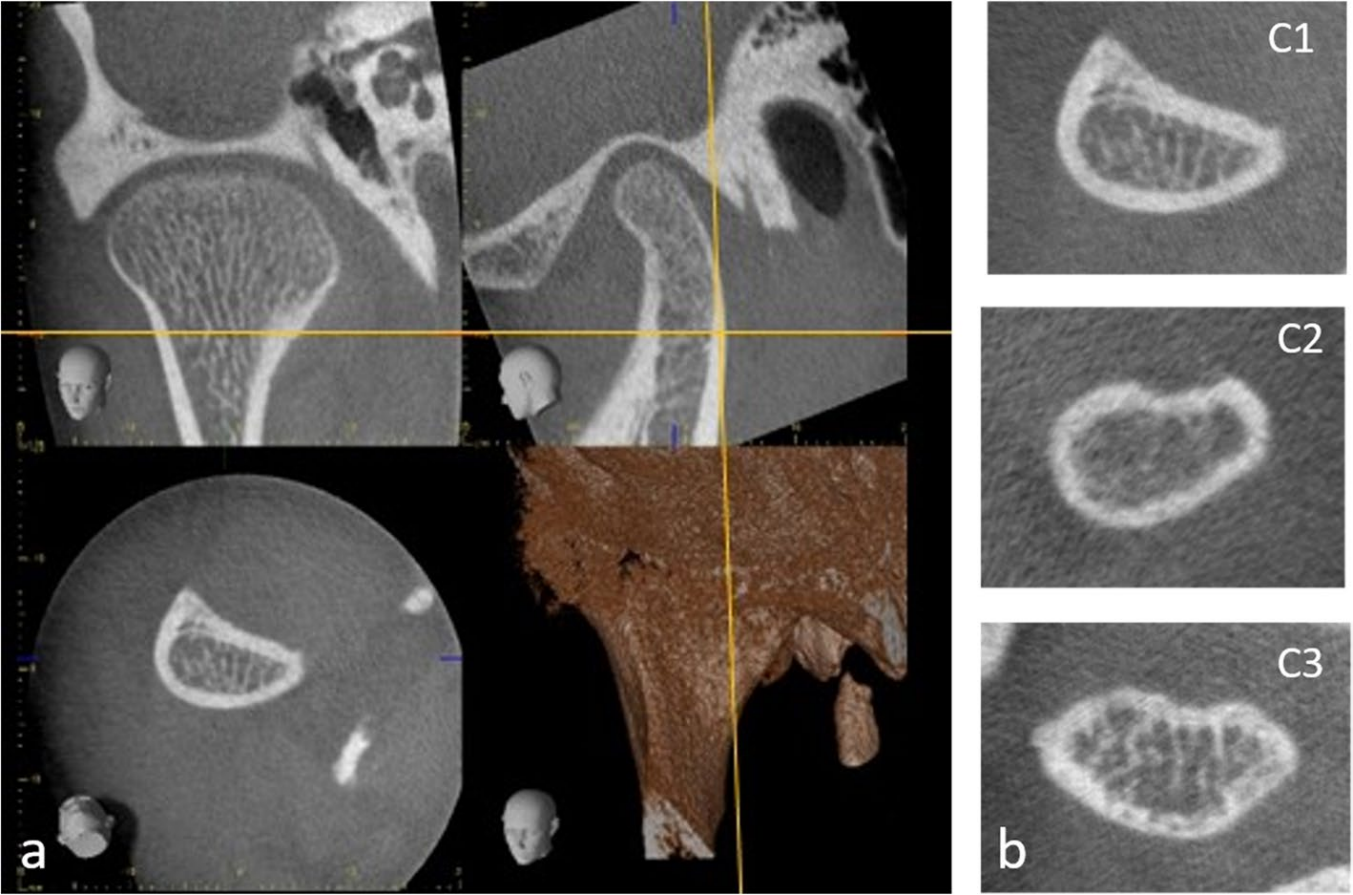
**a**-**b** Multiplanar reconstruction of collum-corrected views (**a**). Examples of axial cross-sectional images from 3 cases representing C1-C3 (**b**)

Multiplanar reconstructions were performed in the following steps: first, the axial and coronal views were adjusted to get an oblique sagittal view trough the mandibular neck. Next, the sagittal view was further orientated to be tangential to the posterior border of the mandibular neck (Fig. 3a). Finally, the cortical bone in the region of the neck was assessed on the axial cross-sectional images and classified into one of the three categories according to Klemetti index/mandibular cortical index [28], which was developed for panoramic radiography, as C1: endosteal margin is even and sharp; C2: endosteal margin presents lacunar resorption or cortical residues on one or both sides, or C3: the cortical layer is clearly porous, with heavy endosteal cortical residues (Fig. 3b).

### Statistical analysis

Continuous data were presented as means (± SD), ordinal data as medians (ranges) and dichotomous data as proportions. Correlations between DXA and DXR were analysed using Pearson correlation coefficient, and statistical significance was tested using Chi squared, or Fisher’s exact tests, as appropriate. For Pearson correlation coefficient, the following interpretation was used: 0.00-0.10: negligible correlation, 0.10-0.39: weak correlation, 0.40-0.69: moderate correlation, 0.70-0.89: strong correlation and 0.90-1.100: very strong correlation [39]. For the assessment of agreement between the BHI SDS as measured by the program BoneXpert and the BHI SDS-equivalent as assessed subjectively, the values were categorized into 5 scores from ≤ -2 to > 2 at intervals of 1. Agreement was analysed using a simple or a weighted Cohen’s Kappa coefficient with 95% confidence intervals. A kappa score of <0.2 was considered poor, 0-0.20 slight, 0.21–0.40 fair, 0.41–0.60 moderate, 0.61–0.80 substantial and 0.81–1.00 almost perfect [40]. A significance level of 0.05 was decided a priori and all the reported *p* values are two-tailed. Statistical analyses were performed using IBM SPSS Statistics, version 28.

### Ethics

The main study (Norwegian JIA Study (NorJIA), NCT number NCT03904459 in www.clinicaltrials.gov.) was approved by the Regional Ethics Committee; REK VEST no 2012/542, and written informed consent was obtained from each participant and/or their caregiver, according to national guidelines. Data was collected and stored according to the General Data Protection Regulation (GDPR).

## Results

A total of 120 children (72 (60.0%) females) with a diagnosis JIA were included, mean age 11.6 years (SD 3.1 years), range 4.9 -16.4 years, of whom 109 also had a CBCT of the TMJs. 

### DXA vs. automated BoneXpert BHI

There was a strong correlation between the absolute values of BHI and BMD for both TBLH (*r* = 0.75, *p*<0.001) and LS (*r* = 0.77, *p*<0.001). The correlation between BoneXpert BHI SDS and DXA TBLH Z-scores was weak to moderate, but significant (0=0.001), with *r*= 0.34, varying from 0.31 to 0.42 between the three study sites. Similar, there was a weak to moderate correlation between the BoneXpert BHI SDS and the DXA LS Z-score, with a correlation coefficient of 0.36 (*p*<0.001), varying between 0.26 and 0.46.

When categorizing the BoneXpert BHI SDS and DXA BMD Z-scores on a 0-5 scale, there was a weak agreement between the two for LS (w-kappa = 0.2 (0.1-0.3), increasing to 0.3 (0.2-0.5) when using quadratic weights) (Table 1). Similar, for TBLH, the agreement was weak with a w-kappa of 0.2 (0.1-0.3) using linear, and 0.3 (0.1-0.5) using quadratic weights. Interrestingly, the agreement was notably higher for one of the three study sites as compared to the two others, particularly for spine assessment, yielding a moderate kappa value of 0.4 – 0.5 (Table 2).

**Table 1:** Agreement between an automated BHI SDS as assessed by BoneXpert, and DXA BMD (L1-L4) Z-score. Both categorized on a 0–5 scale, from < -2 to ≥ 2, at intervals of 1

		Bone Health Index SDS Score (on a 0–5 scale)					Total
		< -2	-2 to -1	-1 to 0	0 to 1	1 to 2	
BMD z-score (L1-L4)	< -2	1	1	0	0	0	2
	-2 to -1	5	8	1	2	0	16
	- 1 to 0	0	9	14	5	6	33
	0 to 1	2	12	17	12	5	47
	1 to 2	1	3	4	6	3	17
	≥ 2	0	0	1	1	0	2
Total		9	33	37	26	14	119

**Table 2:** Agreement between DXA BMD Z-scores and Bone Health Index SDS (categorized on a 0–5 scale) for each of the three study sites

DXA	UNN (*n* = 30) w-Kappa-value (quadratic)	StOlavs (*n* = 52) w-Kappa-value (quadratic)	HUS (*n* = 37) w-Kappa-value (quadratic)
TBLH Z-score vs. BHI SDS score	0.3 (0.4)	0.2 (0.3)	0.2 (0.3)
Spine (L1-L4)-Z-score vs. BHI SDS score	0.4 (0.5)	0.1 (0.2)	0.2 (0.3)

### Radiogrammetry

#### Intra-observer agreement for the assessment of a “Subjective BHI-equivalent”

All 120 children had a radiograph of the left hand. When based on a 0-5 score, the intra-observer agreement for assessment of a “subjective BHI-equivalent” was good, with a w-kappa-value of 0.6 (95%CI= 0.4-0.7).

Of 120 hand radiographs, the cortex was judged to be irregular in 26 examinations on either of the two scorings, yielding a moderate intra-observer agreement (kappa-value 0.5 (95%CI= 0.3-0.7). The zone of provisional calcification (ZPC) could be assessed on 91 hand radiographs in which the physis remained open, with a good intra-observer agreement (w-kappa value 0.6 (95%CI=0.4-0.7) (Table 3).

**Table 3:** Conspicuity of the zone of provisional calcification (ZPC) on the distal radius, assessment based on 120 left hand radiographs in 120 children (72 female) with a diagnosis of JIA, aged 5–16 years of age, of which 91 was assessable

		Not visible	Mildly visible	Moderately visible	Total
Conspicuity of the zone of provisional calcification, radius only	Not visible	3	6	0	9
	Mildly visible	3	53	5	61
	Moderately visible	0	8	13	21
Total		6	67	18	91

Agreement for subjective assessment of low bone mass could not be analysed due to small numbers.

#### BoneXpert BHI vs. a “Subjective BHI equivalent”

Based on a 0-5 score, there was a moderate to good agreement between BHI SDS and subjective BHI SDS (w-kappa = 0.5 (95%CI 0.3-0.6), increasing to 0.6 (0.5-0.7) when using quadratic weights). Seven out of the 9 cases judged to have a BHI SDS of ≤ -2 on BoneXpert were judged to have negative values subjectively, of which all were lower than -1 (Table 4).

**Table 4:** Agreement between BHI SDS as assessed by BoneXpert and a subjective assessment, both based on a 0–5 score, from ≤ -2 to ≥ 2 at intervals of 1. 120 children and adolescents between 5 and 16 years of age

		Bone Health Index SDS Score (on a 0–5 scale)					
		≤ -2	-2 to -1	-1 to 0	0 to 1	1 to 2	Total
Bone Health Index SDS Subjectively (on a 0–5 scale)	< -2	2	2	0	0	0	4
	-2 to -1	5	15	6	4	0	30
	- 1 to 0	1	14	22	8	3	48
	0 to 1	1	2	8	9	4	24
	1 to 2	0	0	1	6	5	12
	≥ 2	0	0	0	0	2	2
Total		9	33	37	27	14	120

#### Associations

There were no significant associations between irregular cortex and BoneXpert BHI score on a 0-5 scale (pearsons chi *p*=0.424), nor ZPC (*p*=0.272) or between irregular cortex and DXA BMD Z-scores on a 0-5 scale (*p*=0.209 and *p*=0.624 for TBLH and LS, respectively).

### CBCT scores – interobserver agreement

109 of the 120 subjects had a CBCT of the TMJs performed, of which 100 left- and 94 right sided scans were acceptable for diagnostics, and thus included in further analysis. Based on a 1-3 scale, 59 out of 94 left TMJ’s were scored as 1 and 31 as score 2 by the first observer vs 87 and 7 by the second observer yielding a poor agreement (kappa value of 0,1).

## Discussion

Children with juvenile idiopathic arthritis are routinely monitored throughout childhood and adolescence, thus, reducing the number of visits and types of examinations is important to minimise the total burdon for these patients. As for the assessment of bone health, we found a strong correlation for the absolute DXR and DXA values (TBLH and LS), but only a weak to moderate, but significant correlation for their z-values.

Categorizing the DXA LS and automated BHI-Z-scores on a 0-5 scale gave a weak to moderate agreement between the two methods, indicating that a hand radiograph might provide an adjunctive tool to DXA when assessing bone health, given thorough calibration is performed. Moreover, assessment of a subjective BHI equivalent performed well as compared to BoneXpert.

Our results compare fairly well to those of a recent systematic review and meta-analysis, concluding that no current imaging modality provides a full evaluation of bone health in children and young adults [27]. The authors identified a total of seven studies comparing DXR and DXA, of which four found a moderate to strong correlation between the two methods (Z-scores, correlation coefficient 0.71), while corresponding figures for DXA vs ultrasound and DXA vs CT were 0.57 and 0.57, respectively [27]. However, in two of the studies the correlation was poor for the Z-scores although the absolute values correlated well. Similarly, our study showed lower correlation, although significant, for the Z-scores as compared to the absolute values. We agree with Shalof and colleagues, suggesting that the lower correlation in part might be due to different reference populations used for DXA and DXR-z-values. Moreover, although all 3 centers used Lunar machines, we speculate that inter-machine differences might have played a role, since the correlation between DXA and DXR appeared to vary across the three study sites.

For the current study, we used bone-age adjusted values for DXA BMD Z-scores in children with growth delay, defined as skeletal age < -2SD for age/gender. Although current understanding is that when interpreting paediatric bone density results, it is preferable to use a size-adjustment method, such as BMAD or a height-adjusted Z-score [11], consensus regarding the most appropriate size-adjustment technique has yet to be established. For this reason the use of age-adjusted BMD is still recommended by ISCD [6, 41].

In sum, we agree with the authors of the systematic review, concluding that DXR may provide results close to those obtained from DXA regarding measuring bone mineral density and might reflect the ability of DXR to be used as a reliable method for evaluating bone density in children and young people [27]. Moreover, the ability of DXR to measure cortical thickness, metacarpal length and width, representing volumetric BMD as opposed to DXA, being a 2D technique, might prove helpful.

Interrestingly, we demonstrated a good intrarater agreement for subjective scoring of a BHI-equivalent and a moderate agreement between the subjective score and the DXR-score, supporting the role of radiographic features as a trigger for more comprehensive bone health testing.

Subjective assessment of an irregular cortex on a 0-1 scale performed well, as did assessment of the zone of provisional ossification on a 0-2 scale. However, we found no significant associations between these two markers and TBLH / LS scores, nor BHI-scores, thus, their clinical value remains unclear.

As for assessment of bone health using CBCT, despite a thorough calibration between observers, the poor interobserver agreement using a 1-3 scale is rather disappointing. An explanation might be that the observers have chosen different images for their assessment, with varying appearances of the bone strudture. The results underscore the need for standardization and further research. The transferability of the classification system suggested by Klemetti is thus unclear [28]. The standardized and multiplanar acquisition of CBCT could open for automated assessment of bone health in the mandible though, but the method has to be tested in a separate study.

Being localised imaging modalities, both CBCT of the TMJs and a hand radiograph might be less suitable for patients with severe involvement of these specific joints. This caveat has to some extent been forseen by the program BoneXpert, rejecting hand images showing abnormal bone morphology such as severe destructive change. On the other hand, decreased BHI and delayed bone maturation has been reported in JIA patients with relatively mild disease, and no correlation with JADAS 10 score, reflecting a generalised poor bone health [42].

## Conclusions

Categorizing the DXA LS and automated DXR-Z-scores on a 0-5 scale gave a weak to moderate agreement between the two methods, indicating that a hand radiograph might provide an adjunctive tool to DXA when assessing bone health, given thorough calibration is performed. Moreover, assessment of a subjective BHI equivalent performed well.

## Data Availability

Data will be available on special request.

## Abbreviations

DXA: Dual-energy X-ray absorptiometry
BHI: Bone health index
BMD: Bone mineral density
CBCT: Cone-beam computed tomography
TBLH: Total body less head
LS: Lumbar spine
SDS: Standard deviation score
ZPC: Zone of provisional calcification
TMJ: Temporomandibular joint
